# Commentary: Questionnaire and behavioral task measures of impulsivity are differentially associated with body mass index: a comprehensive meta-analysis

**DOI:** 10.3389/fpsyg.2017.01222

**Published:** 2017-07-21

**Authors:** Adrian Meule

**Affiliations:** ^1^Department of Psychology, University of Salzburg Salzburg, Austria; ^2^Centre for Cognitive Neuroscience, University of Salzburg Salzburg, Austria

**Keywords:** impulsivity, body mass index, mediation, moderation, moderated mediation

In a recent article, Emery and Levine (in press) report on a meta-analysis examining the relationship between impulsivity measures and body mass index (BMI). They found that impulsivity relates positively, but weakly, to BMI and that behavioral measures of impulsivity produced larger effects than questionnaire measures. Impulsivity domains that assessed disinhibited behaviors, attentional deficits, impulsive decision-making, and cognitive inflexibility produced significant, but small effect sizes. Effect sizes for impulsivity domains related to extraversion/positive emotionality, neuroticism/negative emotionality, and inhibition were not significant. Therefore, these meta-analytic results provide strong support for and are in line with prior observations about the very small correlation between impulsivity and BMI and about the relevance of differentiating between specific impulsivity domains when examining relationships with BMI (Mobbs et al., 2010; Lawyer et al., 2015; Meule and Blechert, 2016; VanderBroek-Stice et al., 2017). This commentary intends to highlight two additional aspects that seem relevant when examining the relationship between impulsivity and BMI. Specifically, it is argued that there are (1) *indirect* effects of impulsivity on BMI through eating behavior (mediation) and (2) *interaction* effects between different impulsivity domains or between impulsivity and eating-related constructs on BMI (moderation). Both of these effects cannot be observed by testing single correlations between impulsivity measures and BMI. In addition, both effects can be found even when impulsivity and BMI appear to be uncorrelated at first glance.

Indirect effects refer to a possible causal chain that describes how (i.e., through which mechanism) an antecedent variable (X) is linked to a consequent variable (Y) through an intermediary variable (i.e., mediator, M). The general association between X and Y without considering M is called the *total effect*. The presence of a total effect (e.g., a significant correlation coefficient between X and Y), however, is not relevant to establishing mediation. That is, it is indeed possible to establish an indirect effect despite no total effect (Zhao et al., 2010; Hayes, 2013; Hayes and Rockwood, in press). For example, it could be that two or more indirect paths carry the effect from X through Y that operate in opposite directions and, thus, can cancel each other out and produce a non-significant total effect (MacKinnon et al., 2000; Hayes, 2009). When a person has an impulsive personality, body fat does not magically increase simply because of that. Instead, impulsivity most likely translates into higher BMI through higher calorie intake (Figure 1A). This is indeed what has been found in recent (yet cross-sectional) studies: there was no total effect of impulsivity on BMI, but an indirect effect of impulsivity on BMI through variables that are associated with increased food consumption (e.g., more frequent and intense food cravings or higher addiction-like eating behavior; Murphy et al., 2014; Meule and Blechert, 2017; VanderBroek-Stice et al., 2017). Future studies may identify additional indirect effects of impulsivity on BMI that may be of opposite direction. For example, impulsivity-associated constructs such as extraversion and sensation seeking have been found to correlate with higher physical activity (Rhodes and Smith, 2006; Leasure and Neighbors, 2014; Wilson and Dishman, 2015; Artese et al., 2017). Thus, physical activity could be a potential mechanism that may link higher impulsivity with lower BMI and, thus, may explain non-significant total effects of impulsivity on BMI.

**Figure 1:**
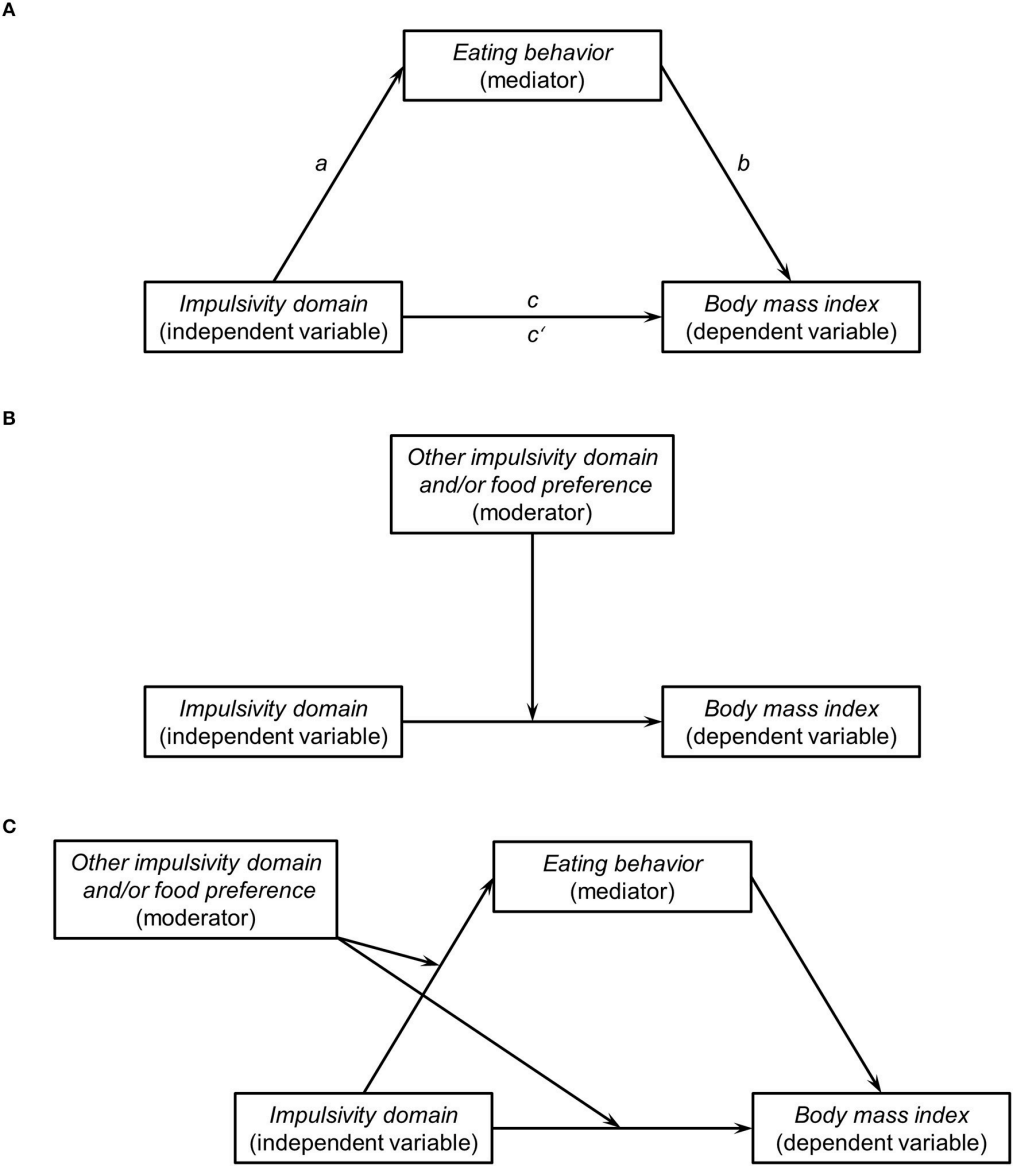
**(A)** Hypothetical simple mediation model that includes an indirect effect of an impulsivity domain on body mass index (BMI) through eating behavior. The variable *impulsivity domain* may represent constructs such as disinhibited behaviors, attentional deficits, impulsive decision-making, or cognitive inflexibility (Emery and Levine, in press). The variable *eating behavior* may represent habitual food consumption (e.g., as measured with a food frequency questionnaire) or constructs such as disinhibited eating, trait food craving, food addiction, binge eating or similar eating styles that are associated with consumption of energy-dense and/or large amounts of food (e.g., Meule and Blechert, 2017; also see Vainik et al., 2015 for a discussion of different eating-related traits that seem to represent the same underlying concept). Path *a* represents the relationship between an impulsivity domain and eating behavior. Path *b* represents the relationship between eating behavior and BMI when controlling for the independent variable. Path *c* represents the relationship between an impulsivity domain and BMI without controlling for the mediating variable (*total effect*). Path *c'* represents the relationship between an impulsivity domain and BMI when controlling for the mediating variable (*direct effect*). The product of *a* × *b* is the indirect effect of an impulsivity domain on BMI through eating behavior. The total effect is the sum of the indirect and the direct effect (*c* = (*a* × *b*) + *c*') and, thus, the presence of a total effect is not a prerequisite for establishing an indirect effect. Therefore, an impulsivity domain may be indirectly associated with BMI through eating behavior, even when the correlation coefficient between that impulsivity domain and BMI (i.e., the total effect) is not statistically significant. **(B)** Hypothetical moderation model, in which the relationship between an impulsivity domain and BMI depends on a moderating variable. This moderating variable may be another impulsivity domain and/or may be an eating-related variable such as preference for high-calorie foods. Therefore, an impulsivity domain may be associated with BMI as a function of a moderating variable, even when the correlation coefficient between that impulsivity domain and BMI is not statistically significant. **(C)** Hypothetical moderated mediation model, in which the moderating variable in **(B)** not only moderates the total effect of an impulsivity domain on BMI, but also moderates the indirect effect of an impulsivity domain on BMI. For example, there may be an indirect effect of an impulsivity domain on BMI through eating behavior, but only at high levels on another impulsivity domain and/or only in individuals that demonstrate a high preference for high-calorie foods. These are just a few examples of how and under which circumstances a high impulsivity may translate into higher BMI as (1) all paths (*a, b*, and *c*) can potentially be moderated (and by different variables), (2) paths *a* and *b* may include additional mediators that link the independent variable with the mediator and the mediator with the dependent variable (*serial mediation*), and (3) there may be several mediators that act simultaneously *(parallel mediation)*.

Interaction effects refer to the question about when (i.e., under which circumstances) or for whom an antecedent variable is linked to a consequent variable, contingent on a moderating variable. For example, BMI (or calorie intake) may be particularly high when a person scores high on more than one impulsivity domain (Figure 1B). Furthermore, having an impulsive personality may only lead to weight gain when combined with a strong preference for high-calorie foods (Figure 1B). This has indeed be found in recent studies: one impulsivity domain (*attentional impulsivity* as measured with the Barratt Impulsiveness Scale) was particularly associated with higher calorie intake, disinhibited eating behaviors, or body fat when individuals also had higher scores on another impulsivity domain (*motor impulsivity* as measured with the Barratt Impulsiveness Scale), but not when individuals had lower scores on this domain (Kakoschke et al., 2015; Meule and Platte, 2015; Meule et al., 2017). Higher impulsivity predicted higher unhealthy food consumption in the laboratory, but only when participants also demonstrated high food reward sensitivity or implicit preference for high-calorie foods (Friese and Hofmann, 2009; Hofmann et al., 2009; Appelhans et al., 2011). In two longitudinal studies, higher impulsivity prospectively predicted weight gain, but only in participants who showed a high preference or attentional bias for high-calorie foods (Nederkoorn et al., 2010; Meule and Platte, 2016).

In conclusion, the meta-analytic findings by Emery and Levine (in press) demonstrate that the total effect of impulsivity on BMI is very small and that the size of this total effect differs depending on the specific impulsivity domain that is investigated. Because of its small effect size, the relationship between impulsivity and BMI is likely to be non-significant in underpowered studies and researchers will conclude that impulsivity and BMI were unrelated in their respective investigation. As has been highlighted in this commentary, however, there are likely indirect effects of impulsivity on BMI through eating behavior-related variables (Figure 1A) and interaction effects between different impulsivity domains or between impulsivity and eating behavior-related variables (Figure 1B), even when there is no directly observable, significant correlation between the respective impulsivity measure and BMI. Such mediation and moderation effects can also be integrated into one moderated mediation model (Figure 1C; e.g., Meule et al., 2016). Therefore, researchers are encouraged to conduct such analyses, particularly when a correlation between impulsivity and BMI is absent or small. Ideally, testing for such effects will become a default analysis strategy, which will ultimately contribute to generating a comprehensive model of *how* and *when* or *for whom* an impulsive personality poses a risk for becoming overweight or obese.

## Author contributions

The author confirms being the sole contributor of this work and approved it for publication.

### Conflict of interest statement

The author declares that the research was conducted in the absence of any commercial or financial relationships that could be construed as a potential conflict of interest.
